# PPAR-pan activation induces hepatic oxidative stress and lipidomic remodelling

**DOI:** 10.1016/j.freeradbiomed.2015.11.033

**Published:** 2016-06

**Authors:** Zsuzsanna Ament, James A. West, Elizabeth Stanley, Tom Ashmore, Lee D. Roberts, Jayne Wright, Andrew W. Nicholls, Julian L. Griffin

**Affiliations:** a Medical Research Council, Human Nutrition Research, Elsie Widdowson Laboratory, 120 Fulbourn Road, Cambridge CB1 9NL, UK; b The Department of Biochemistry, University of Cambridge, 80 Tennis Court Road, Cambridge CB2 1GA, UK; c The Cambridge Systems Biology Centre (CSBC), University of Cambridge, Cambridge CB2 1QR, UK; d Jayne Wright Ltd., Underhill House, Ledbury, Herefordshire HR8 2QR, UK; e GlaxoSmithKline, Investigative Preclinical Toxicology, Park Road, Ware SG12 0DP, UK

**Keywords:** Peroxisome proliferator-activated receptors, Lipidomics, Metabolomics, β-Oxidation, Eicosanoids

## Abstract

The peroxisome proliferator-activated receptors (PPARs) are ligand activated nuclear receptors that regulate cellular homoeostasis and metabolism. PPARs control the expression of genes involved in fatty-acid and lipid metabolism. Despite evidence showing beneficial effects of their activation in the treatment of metabolic diseases, particularly dyslipidaemias and type 2 diabetes, PPAR agonists have also been associated with a variety of side effects and adverse pathological changes. Agonists have been developed that simultaneously activate the three PPAR receptors (PPARα, γ and δ) in the hope that the beneficial effects can be harnessed while avoiding some of the negative side effects.

In this study, the hepatic effects of a discontinued PPAR-pan agonist (a triple agonist of PPAR-α, -γ, and -δ), was investigated after dietary treatment of male Sprague–Dawley (SD) rats. The agonist induced liver enlargement in conjunction with metabolomic and lipidomic remodelling. Increased concentrations of several metabolites related to processes of oxidation, such as oxo-methionine, methyl-cytosine and adenosyl-methionine indicated increased stress and immune status. These changes are reflected in lipidomic changes, and increased energy demands as determined by free fatty acid (decreased 18:3 *n*−3, 20:5 *n*−3 and increased ratios of *n*−6/*n*−3 fatty acids) triacylglycerol, phospholipid (decreased and increased bulk changes respectively) and eicosanoid content (increases in PGB2 and 15-deoxy PGJ2). We conclude that the investigated PPAR agonist, GW625019, induces liver enlargement, accompanied by lipidomic remodelling, oxidative stress and increases in several pro-inflammatory eicosanoids. This suggests that such pathways should be monitored in the drug development process and also outline how PPAR agonists induce liver proliferation.

## Highlights

• The investigated PPAR-pan agonist increased oxidative damage in the rat liver.
• Metabolomics can be used to follow the dose response nature of PPAR-pan agonists.
• Metabolomics is a versatile tool in following oxidative stress.

## 1 Introduction

Peroxisome proliferator-activated receptors, PPAR-α, PPAR-γ, and PPAR-β/δ (PPAR-δ) are differentially expressed in various tissues mediating numerous metabolic processes and globally regulating systemic metabolism in mammals [1]. PPAR-α and PPAR-δ have important roles in regulating β-oxidation in the liver and skeletal muscle, respectively, while PPAR-γ is involved in the sequestration of triglycerides to adipose tissue and overall adipose tissue expandability [2], [3]. The dominant effects of the three PPARs are to a degree tissue specific. PPAR-α is mainly expressed in the liver, PPAR-γ is primarily expressed in adipose tissue whilst PPAR-δ is ubiquitously expressed and abundant in most tissues [4], [5], [6], [7]. However, all three receptors are present in the adult rat liver [4].

PPAR-α agonists have been used to reduce plasma triglyceride, reduce low density lipoprotein cholesterol and increase high density lipoprotein levels [8], [9]. Their lipid lowering effects are in part brought about by increased β-oxidation in the liver and skeletal muscle [10]. In addition PPAR-α in part regulates liver metabolism by increasing glycolysis and reducing gluconeogenesis in the liver [11] and agonists have been shown to have potent ability to decrease glucose concentrations in blood plasma [12]. PPAR-γ agonists improve insulin sensitivity [13], [14] in part by stimulating lipid uptake as well as de novo lipogenesis by adipocytes. PPAR-γ agonists also cause increased glucose utilisation and decreased serum glucose levels without the need for increased insulin secretion. In normal liver, PPAR-γ is expressed at a very low level but it has been reported to reach functionally significant expression levels in steatotic liver [15]. PPAR-δ agonists have also been shown to improve insulin resistance by increasing fatty acid oxidation and reduce serum glucose levels [13]. Adverse findings including apoptosis, inflammation and potentially carcinogenesis have all been reported as a consequence of PPAR-δ activation. There are however confounding reports regarding whether PPAR-δ ligands promote or inhibit these effects depending on the experimental conditions used [16], [17].

One of the proposed strategies for improving lipid metabolism in metabolic diseases is to use a molecule which can simultaneously activate two or three PPAR receptors. It was hoped that such PPAR-pan agonists would increase hepatic fatty acid oxidation by stimulating PPAR-α and PPAR- δ, and further improve insulin sensitivity by stimulating all three PPARs, and thus favourably influence conditions associated with the metabolic syndrome and type two diabetes mellitus (T2DM), whilst the negative effects, such as increased adiposity caused by PPAR-γ leading to weight gain would be negated by increased fat oxidation promoted by PPAR-α and PPAR-δ.

The agonist was designed to simultaneously activate all three isoforms of the PPARs to treat the metabolic syndrome. However, this compound was withdrawn from development following the induction of increased liver weight and myopathy in male Sprague–Dawley (SD) rats. Although many of the metabolic regulatory roles of PPARs have been clarified, the reasons for their adverse effects have remained elusive and currently, no triple (-pan) or dual activators are on the market. Furthermore, by better defining these adverse effects we may be able to screen for PPAR agonists that should be excluded early in the drug safety assessment process.

Mechanism underlying the toxicity of PPAR-pan activation most likely involve lipid-mediated processes, however, these are yet to be defined. With a better understanding of the pathophysiology that accompanies PPAR-pan agonists, it could become possible to target activation of the beneficial responses of PPAR signalling without the pathological effects. In the present study a comprehensive array of mass spectrometric approaches were used in conjunction with multivariate statistics to define the metabolic effects of a PPAR-pan agonist known to be associated with liver enlargement and toxicity. Our results identify a dose responsive decrease in triacylglycerol (TG) and increase in acyl-carnitine and phospholipid concentrations, reflecting increased β-oxidation and cell and/or organelle proliferation, respectively. These responses are likely associated with targeting PPAR-α and PPAR-δ. There were also increases in metabolites associated with processes of oxidation, including oxo-methionine, methyl-cytosine and adenosyl-methionine, further contributing to undesirable side effects and potential DNA damage. In addition, dose dependent increases were measured in the concentrations of inflammatory prostaglandins (PGs) 15d-PGJ2 and PGB2. Furthermore, we hypothesise that the increased levels of inflammatory eicosanoids, which themselves can act as endogenous PPAR activators [18], potentially generate a positive feedback loop.

## 2 Materials and methods

### 2.1 Animal experiments and study design

All animal studies were ethically reviewed and carried out in accordance with Animals (Scientific Procedures) Act 1986 and the GSK Policy on the Care, Welfare and Treatment of Animals.

PPAR-pan activator was administered to male Sprague–Dawley rats (Crl:CD (SD) strain), 12 animals per group, by daily oral gavage at 30, 100, 300, 1000 mg/kg/day for 13 weeks. A separate satellite group of animals (6 per group) were kept for a 4 week treatment free period in the control, intermediate 2 (300 mg/kg/day) and high (1000 mg/kg/day) dose groups (Table 1). According to the principles of the 3Rs (replacement, reduction and refinement) recovery animals were not used for the low and for the intermediate 1 dose groups.

### 2.2 Collection of samples

Blood and urine samples of all animals were collected in weeks 4, 13 and 18. At necropsy (at the end of dosing or following the recovery period), tissue samples were collected following an overdose of anaesthetic (halothane Ph. Eur. Vapour). Samples of the liver were immediately removed, weighed, and sections snap-frozen in liquid nitrogen. Samples were maintained at −80 °C until further analysis. Tissue samples from all organ systems were fixed in neutral buffered formalin and process to slides. They were examined by light microscopy for evidence of pathological changes.

### 2.3 Analytical measurements

#### 2.3.1 Extraction procedure

Briefly, methanol: chloroform solution (2:1, 600 µL) was added to approximately 50 mg of frozen tissue and homogenised with a tissue lyser. Chloroform and water (200 µL each) was added, samples were sonicated for 15 min and centrifuged (13,500 rpm, 20 min). The resulting aqueous and organic layers were separated and the extraction procedure was repeated. Samples were dried under nitrogen before processing for gas-chromatography–mass spectrometry (GC–MS) and liquid chromatography–mass spectrometry (LC–MS). GC–MS, and LC–MS/MS methods for lipid extraction and analysis were carried out according to methods previously described [19], [20].

### 2.4 GC–MS analysis of fatty acid methyl esters (FAMEs)

For GC–MS, the resulting organic fractions from the chloroform: methanol extraction were used. Samples were reconstituted in 1 mL of methanol:chloroform 2:1 and a fifth of each sample (200 µL) was transferred to a 3 mL glass vial. The 200 µL aliquots were dried under nitrogen before being derivatised with a methylating agent which forms fatty acid methyl esters (FAMEs) of carboxylic acids. Chloroform:methanol (1:1, 100 μl), boron trifluoride in methanol (10 %, 125 μl) and 150 µL d-25-tridecanoic acid (200 µM in chloroform) were added to the dried extracts. Samples were vortex mixed and heated to 80 °C for 90 min. After cooling, 300 µL water and 600 µL hexane were added. The samples were vortex mixed, the lower aqueous layer was removed and the remaining organic layer dried under nitrogen. The samples were reconstituted in 150 μl hexane and transferred to autosampler vials prior to analysis using a Trace GC Ultra coupled to a DSQ II single-quadrupole mass spectrometer (Thermo Scientific, Hemel Hempstead, Hertfordshire). Samples were injected onto a Zebron™ ZB-WAX column (100% polyethylene glycol 30 m×0.25 mm ID, 0.25 µm film thickness). The injector temperature was 230 °C and the flow rate of helium was 1.2 mL/min. The column was held at 60 °C for 2 min, after which the temperature was increased to 150 °C at a rate of 15 °C/min, and finally increased to 240 °C at a rate of 2.5 °C/min. The transfer line temperature was maintained at 240 °C, while the ion source was at 250 °C, operating at 70 eV for electron ionisation (EI). The detector was initiated after 240 s, and full scan spectra were collected over a range of 50–650 *m/z*.

### 2.5 Open profiling LC–MS/MS analysis of intact lipids

For LC–MS/MS analysis of lipids, the organic fractions of the stock chloroform:methanol extraction were used. A 10 µL aliquot, comprising one hundredth of the organic fraction, was diluted into 90 µL of methanol–chloroform (2:1) containing 20 µM 1,2-diheptadecanoyl-sn-glycero-3-phosphocholine (PC (17:0/17:0)) (Avanti Polar Lipids Inc., Alabaster, Alabama, US) The instrumentation comprised a Xevo G2 Quadrupole Time of Flight (QToF) mass spectrometer with a Z-spray electrospray source (Waters Ltd., Elstree, Hertfordshire, UK) coupled to an ACQUITY Ultra Performance Liquid Chromatography (UPLC) system (Waters Ltd., Elstree, Hertfordshire, UK). Separation of species was achieved using an Acquity CSH C18, 1.7 µm (2.1×100 mm) column (Waters Ltd., Elstree, Hertfordshire, UK). Mobile phase A consisted of 10 mM ammonium formate in acetonitrile:water (6:4), whilst mobile phase B contained 10 mM ammonium formate in isopropanol:acetonitrile (9:1). The concentration of mobile phase B was increased from 40% to 100% over 18 min, then equilibrated to 40% B for 2 min at a flow rate of 0.4 mL/min. The electrospray source was operated in positive ion mode with the source temperature set at 80 °C and a cone gas flow of 100 L/h. The desolvation gas temperature was 250 °C and the nebuliser gas flow rate was set at 700 L/h. The capillary voltage was 3 kV and the cone voltage 50 V. Mass spectrometric data were collected from 50 to 1200 *m/z* in profiling scan mode. For structural elucidation, identification and confirmation of the lipid species present, data-dependent acquisition (DDA) experiments were conducted using pooled samples with five separate scan events. First, the mass spectrometer was set to perform a full-scan, after which an MS/MS scan on the most intense, the second, third and fourth most intense ions was carried out. MS/MS was obtained at a scan rate of 0.6 s with a 0.05 s interscan delay and collision ramps from 20 to 40 eV and 25 to 50 eV. A dynamic exclusion window was set to 0.2 s. The MS scanning was switched to MS/MS acquisition when the threshold value of 1000 intensity/s was exceeded. The MS/MS returned to scanning mode when the signal of the total ion current (TIC) fell below 1000 intensity/s or after 1 s.

Data were processed using MarkerLynx™ within the software suite MassLynx™ (version 4.1) by Waters Ltd. (Elstree, Hertfordshire, UK). Collection Parameters were set with a mass window of 0.05 Da and retention time window of 0.2 min. Data were automatically deisotoped and normalised to the intensity of the internal standard. Tentative identifications were made based on exact mass information using the Lipid Maps structure database (Lipid Maps, La Jolla, California, US). DDA data was used to confirm or reject tentative IDs.

### 2.6 Targeted analysis of aqueous metabolites

For LC–MS/MS analysis aqueous phase metabolites resulting from the chloroform:methanol extractions were used. The entire fraction was dissolved in 300 µl of 70:30 acetonitrile:water containing 20 µM universally ^13^C- and ^15^N- labelled glutamate. Samples were vortex mixed, sonicated, centrifuged, (17,000×*g*, 5 min) pipetted into auto-sampler vials and analysed using an AB Sciex 5500 Qtrap mass spectrometer (AB Sciex UK Limited, Warrington, Cheshire) coupled to a SIL20-A LC system (Shimadzu Corp., Kyoto, Japan). Mobile phase A consisted of 100 mM ammonium acetate, mobile phase B was acetonitrile, and the flow rate was 0.3 mL/min. Two microliters of each sample was injected, and analytes separated using a 100 mm ZIC-HILIC column with 2.1 mm ID and 3.5 µm particle size (Sequant, Umeå, Sweden). A linear gradient was used, starting at 20% A for 2 min, followed by an increase to 50% A over 10 min, and finally a 3 min re-equilibration. Metabolites of interest were measured in positive ionisation mode with unscheduled multiple reaction monitoring events (MRMs) (Supplementary Table 2), using a source temperature of 500 °C, an ion spray voltage of 4.5 kV and a dwell time of 50 ms. Peaks were integrated by the Quantitation Wizard within Analyst™ version 1.6 by AB Sciex Ltd. (Warrington, Cheshire, UK) and normalised against wet tissue weight and to the intensity of the internal standards.

### 2.7 Analysis of acyl-carnitines

Acyl-carnitines were measured according to the method described by Roberts et al. [21]. Briefly, 100 µL internal standard solution mix (1.63 µM [D9] free carnitine, 0.3 µM [D3] acetyl carnitine, 0.06 µM [D3] propionyl-carnitine, 0.06 µM [D3] butyryl-carnitine, 0.06 µM [D9] isovarelyl-carnitine, 0.06 µM [D3] octanoyl-carnitine, 0.06 µM [D9] myristoyl-carnitine, and 0.12 µM [D3] palmitoyl-carnitine, Cambridge Isotope Laboratories, Andover, MA, USA) was added to 40 µL of the organic fraction of the methanol:chloroform extraction and the resulting mixture were dried down under nitrogen and derivatised with 100 µL of 3 M butanolic-HCl (Sigma-Aldrich, Louis, Missouri, USA). Samples were evaporated under nitrogen, re-constituted and sonicated in 4:1 acetonitrile: 0.1% formic acid in water before transferring them to autosampler vials. Samples were analysed using an AB Sciex 5500 QTRAP mass spectrometer (AB Sciex UK Limited, Warrington, Cheshire) coupled to an Acquity UPLC system. Mobile phase A consisted of 0.1% formic acid in water, while mobile phase B was acetonitrile. Two microliters of each sample was injected onto a Synergi Polar RP phenyl ether column (100 mm×2.1 mm, 2.5 µm) supplied by Phenomenex (Macclesfield, Cheshire, UK). The analytical gradient started at 30% B, followed by a linear increase to 100% B over 3 min. The gradient was then held at 100% B for 5 min, after which it was returned to the re-equilibration level of 30% B for 2 min. A flow rate of 0.5 mL/min was used throughout. Data were analysed using the Quantitation Wizard within Analyst™ version 1.6 by AB Sciex Ltd. (Warrington, Cheshire, UK) and normalised against wet tissue weight and to the intensity of the internal standard.

### 2.8 Extraction and analysis of eicosanoids

Eicosanoids were extracted using solid phase extraction (SPE) Waters Oasis-HLB cartridges (Waters Ltd., Elstree, Hertfordshire, UK) as described by Roberts and colleagues [21]. SPE columns were washed with ethyl acetate (2 mL), methanol (2×2 mL), and 15% methanol with 0.1% acetic acid (2 mL). Approximately 100 mg liver tissue samples were homogenised on a TissueLyser (Qiagen Ltd., Manchester, UK; 10 min at 30 Hz) in 1.5 mL 15% methanol with 0.1% acetic acid. The samples were centrifuged (17,000×*g*, 2 min) and the supernatant loaded onto the SPE columns. Cartridges were washed with 1 mL 15% methanol with 0.1% acetic acid. Analytes of interest were eluted with 0.5 mL of methanol followed by 1 mL of ethyl acetate and immediately dried under nitrogen. Samples were finally reconstituted in 40 µL methanol containing 70 nM PGE2-d4 internal standard and transferred to autosampler vials. Analysis was performed using a 4000 QTRAP mass spectrometer (AB Sciex UK Limited, Warrington, Cheshire) coupled to an Acquity ultra performance liquid chromatography (UPLC) system (Waters Corp., Milford, MA). The autosampler was maintained at 4 °C, LC separation was achieved using a Luna, 3 μm particle size, 150×2 mm column (Phenomenex Macclesfield, Cheshire, UK). The gradient of mobile phase A (0.1% acetic acid in water) and B (0.1% acetic acid in acetonitrile:methanol 80:20) is detailed in Supplementary Table 3. The flow rate was held at 0.4 mL/min. Metabolites of interest were measured in negative ionisation mode with unscheduled multiple reaction monitoring events (MRMs) (Supplementary Table 4). Peaks were integrated by the Quantitation Wizard within Analyst™ version 1.6 by AB Sciex Ltd. (Warrington, Cheshire, UK) and normalised against wet tissue weight and to the intensity of the internal standard.

### 2.9 Enzyme-linked immunosorbent assay (ELISA)

An ELISA assay was carried out to profile 4 cytokines: tumour necrosis factor-alpha (TNF-α), interleukin 6 (Il-6), interferon production regulator (IFNr) and interleukin-1 alpha (IL-1α) (Signosis Inc, Santa Clara, CA). Approximately 50 mg of liver tissue was homogenised in 1 mL of radio-immunoprecipitation assay (RIPA) buffer containing cOmplete, mini protease inhibitor tablets (Sigma-Aldrich Co., Dorset, UK) and phenylmethanesulphonil fluoride (1 mM). The homogenised samples were sonicated and centrifuged (15,000 g for 20 min at 4 °C). Protein quantification of the supernatant was carried out using Pierce BCA protein assay kit (Thermo Fisher Scientific Inc., Paisley, UK) and samples were diluted to approximately 0.4 μg/μL total protein content. The ELISA protocol was carried out according to the manufacturer's instructions and absorbance was measured spectrophotometrically at 450 nm using a TCAN NanoQuant Infinite M200Pro spectrometer (Tecan Group Ltd., Männedorf, Switzerland).

### 2.10 RNA purification

Total RNA was purified from frozen rat liver using an RNeasy Mini Kit (Qiagen Ltd. Manchester, UK) according to manufacturer's specifications. Approximately 50 mg tissue, 1 mL QIAzol and a 5 mm metal bead were added to a 2 mL microcentrifuge tube. The samples were lysed and homogenised in a TissueLyzer (Qiagen Ltd. Manchester, UK), and subsequently incubated at room temperature for 5 min. Chloroform (200 µL) was added to each sample, vortexed thoroughly and incubated at room temperature for 3 min. The samples were centrifuged at 4 °C and 12,000×*g* for 15 min. The aqueous (upper) fraction containing RNA was transferred to a new tube and 1 volume of 70% ethanol was added. The samples were vortexed, added to spin columns and centrifuged at 8000×*g* for 15 s to bind RNA to the membrane, with the remainder of the procedure continuing as described in the RNeasy RNA purification kit. RNA concentration was quantified at 260 nm using a NanoDrop 100 (Thermo Fisher Scientific Inc., Paisley, UK).

### 2.11 Reverse transcription and qPCR

Genomic DNA contamination was first eliminated using the RT2 First Strand Kit (Qiagen Ltd. Manchester, UK). Briefly, 400–500 ng RNA, diluted as required into a final volume of 8 µL using RNase-free water, and 2 µL Buffer GE were added to a 0.5 mL PCR tube. The samples were then heated to 42 °C for 5 min and cooled to 4 °C for 10 min in a PTC-200 Thermocycler (MJ Research). Complimentary DNA was produced from the gDNA elimination reaction using the above RT2 First Strand Kit by adding 4 µL 5× Buffer BC3, 1 µL Control P2, 2 µL Buffer RE3 and 3 µL RNase-free water. The samples were incubated at 42 °C for 15 min followed by 95 °C for 5 min in the thermocycler and the produced cDNA produced frozen at −20 °C. For analysis of steady-state mRNA levels, relative abundance of transcripts of interest was assessed by quantitative-PCR in RT2 SYBRgreen Mastermix (Qiagen Ltd. Manchester, UK) with a StepOnePlus detection system (Applied Biosciences). RT2 primer assays for rat Sod2, Gss, Gstk1, Lonp1, Tnf and Il1α were obtained from QIAgen. Thermocycler parameters were as follows: initial incubation, 95 °C for 10 min; with 40 cycles of both elongation, 15 s at 95 °C; and cooling, 1 min at 60 °C. Expression levels were normalised to Rn18s using the ∆∆CT method, and subsequently to samples of the control group to give fold-changes.

### 2.12 Multivariate statistical analysis

For multivariate data processing the software package SIMCA 13 (Umetrics AB, Malmö, Sweden) was used and partial least squares discriminant analysis (PLS-DA) was employed. Model validation was performed by cross validation using 100 random permutations of the PLS-DA models.

## 3 Results and discussion

### 3.1 PPAR-pan treatment causes classic dose dependent response

**Fig. 1:**
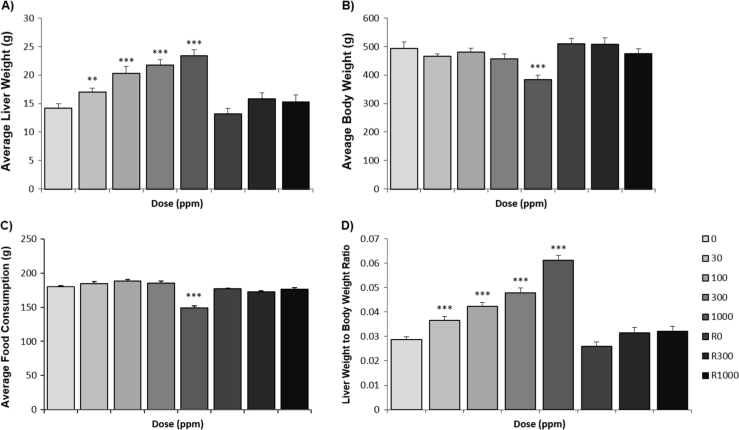
Summary of changes in body, liver and liver to body weight ratios. Changes in body weight (A) liver weight (B) and liver to body weight ratios (C) during the experiment including the recovery period. (Results from the recovery period are marked with the suffix’ ‘R’.) Variables were analysed using one-way analysis of variance (ANOVA). *p<0.05, **p<0.01, ***p<0.001.

A discontinued PPAR-pan activator [22] was administered to male Sprague–Dawley rats (Crl: CD (SD) strain), 12 animals per group, by daily oral gavage at 30, 100, 300, 1000 mg/kg/day for 13 weeks (Table 1). Food intake was normal in all dose groups except in the high dose group, where food intake was reduced at week 12 by 20%. The most pronounced effect on body weight was observed in animals given 1000 mg/kg/day with a total body weight loss of 22% (*p*<0.001). Liver weights increased with dose by 20%, 44%, 54%, and 65% (*p*<0.001) at 30, 100, 300 and 1000 mg/kg/day, respectively, compared to controls. After the withdrawal of treatment in the recovery groups, animals gained total weight during the 4 week recovery period and the liver weights decreased back to control values (Fig. 1). The, reductions in food consumption noted at 1000 mg/kg/day in Week 12 correlate with the observed reduction in bodyweight, suggesting that weight loss is associated with decreased food intake, as well as the PPAR-pan agonist activity, increasing oxidation of carbohydrates and fatty acids.

Plasma aspartate aminotransferase (AST), plasma alkaline phosphatase (ALP), and albumin concentrations [23] were all increased with treatment reflecting liver damage compared to concurrent controls in animals given doses of 300 or 1000 mg/kg/day, respectively. Other changes include increased alanine aminotransferase (ALT) at the highest dose level and total bilirubin levels (Bil) in all treatment groups (Table 2).

Creatine kinase (CK) concentrations were increased in animals at the 1000 mg/kg/day dose (*p*<0.001) presumably reflecting muscle myopathy. There were no clear dose response variations in the urinalysis parameters although an increase in glucose concentrations (*p*<0.05) was observed at the highest dose level by week 12.

Microscopic observations detected hepatocyte hypertrophy, which increased in severity in the high dose groups. This is a well-recognised response to the proliferation of sub-cellular organelles, including peroxisomes. In addition, the heart and muscles (skeletal, soleus and gastrocnemius) showed evidence of myopathy, increasing in severity with dose. There was evidence of fat mobilisation as seen by decreased adipocyte size (abdominal fat), macrovesiculation in brown fat and microevesiculation in the adrenal glands (Supplementary Table 1). Following withdrawal of the compound the incidence and severity of both histological and clinical chemistry changes decreased, suggesting that they were transitory in nature.

### 3.2 Lipidomic remodelling indicates inflammation in a dose dependent manner in response to PPAR-pan agonist

**Fig. 2:**
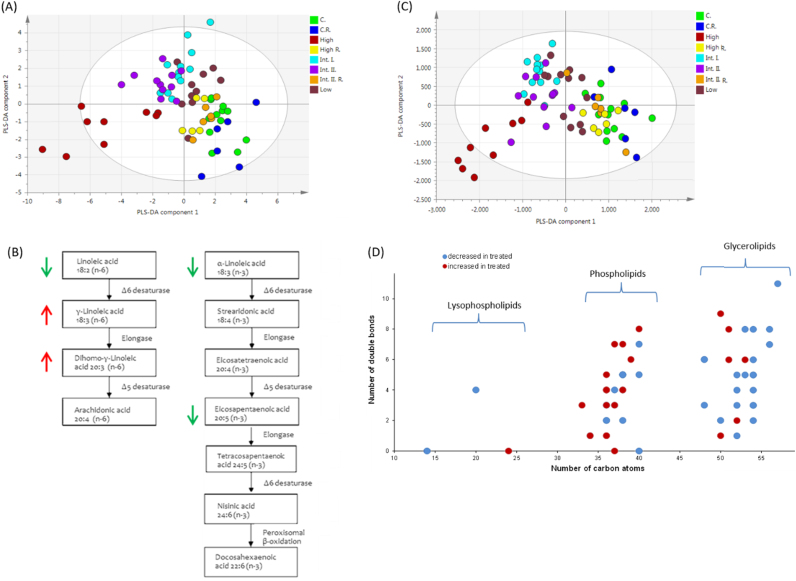
Overview of gas chromatography–mass spectrometry (GC–MS) and liquid chromatography–mass spectrometry (LC–MS) data from the lipid extracts of rat liver tissue samples treated with the PPAR-pan agonist GW625019. (A) Partial least squares-discriminant analysis (PLS-DA) scores plot shows total fatty acid data from lipid extracts of liver tissue samples with clear clustering associated with dose of the agonist. (B) Metabolic changes caused by PPAR-pan agonist GW625019 are increased γ-linoleic acid (GLA) and dihomo-γ-linoleic acid (DGLA). Linoleic acid (LA), α-linoleic acid (ALA) and eicosapentaenoic acids (EPA) concentration were decreased. Arrows show the direction of changes in lipid concentrations relative to control samples in red (increased) and green (decreased) respectively. (C) PLS-DA scores plot of LC–MS intact lipid data (D) and an overview of the chain length and the saturation of changing lipids. Red circles represent the compounds which have increased, whereas blue circles show decreased lipid species. Treatment abbreviations: C., control; Int. I., intermediate 1; Int. II., intermediate 2; R., recovery.

Hepatic total fatty acids were measured by GC–MS (Supplementary Data 1 [24]). For an overview of total fatty acid changes multivariate data analysis was performed and a PLS-DA model was constructed to assess the dose response (*R*^2^=73%, *Q*^2^=45%). The highest dose group clustered separately while there was a linear relationship going from the low to intermediate 2 dose levels showing a dose dependent change in the metabolite concentrations. Furthermore, the recovery groups from both the two intermediate and the high dose groups clustered with the control samples (Fig. 2A). Fatty acids were analysed by one way ANOVA with Bonferroni post-tests. The investigated PPAR-pan agonist caused a decrease in relative concentrations of the main *n*−3 fatty acids α-linoleic acid (ALA,18:3 *n*−3) (*p*<0.01 at 30 mg/kg/day and *p*<0.001 at dose levels 100, 300 and 1000 mg/kg/day) and eicosapentaenoic acid (EPA, 20:5 *n*−3) (*p*<0.05) at the two intermediate dose levels (100 and 300 mg/kg/day). The third main *n*−3 fatty acid docosahexaenoic acid (DHEA, 22:6 *n*−3) showed no significant decrease. However, the *n*−3 or omega-3 index (EPA+DHEA expressed as the percentage of total identified fatty acids) decreased (*p*<0.01) at the highest dose level. Ratios of *n*−6/*n*−3 fatty acids increased at all dose levels (*p*<0.001). Arachidonic acid (AA *n*−6) and eicosapentaenoic acid (EPA *n*−3) are parent compounds for the production of inflammatory mediators often with opposing metabolic and functional properties. The class of *n*−3 fatty acids are reported to have effects preventing proliferation and initiating apoptosis [25], [26] whereas *n*−6 fatty acids are thought to have opposing effects through eicosanoids formed by AA which contribute to inflammation and cell proliferation [25], [26].

Relative concentrations of γ-linoleic acid (GLA, 18:3 *n*−6) and dihomo-γ-linoleic acid (DGLA, 20:3 *n*−6) were increased whereas linoleic acid (LA, 18:2 *n*−6) concentrations decreased at all dose levels (Fig. 2B). Linoleic acid (LA, *n*−6) is metabolised by Δ6 desaturase to form gamma-linoleic acid (GLA *n*−6) which is rapidly elongated to DGLA. DGLA is further desaturated to AA by Δ5 desaturase but this enzyme has limited activity in rodents and this caused DGLA to accumulate more rapidly than AA in the present study when the pathway was activated by the PPAR-pan agonist. Both the Δ5 desaturase and the Δ6 desaturase are transcriptional targets of PPAR-α and PPAR-δ [27], [28].

Monounsaturated fatty acid (MUFA) concentrations increased at the highest dose (*p*<0.01), and polyunsaturated (PUFA) concentrations decreased at the intermediate dose 2 (*p*<0.01) and the highest dose (*p*<0.001) levels. In addition, at the highest dose, saturated fatty acids (SFA) myristic acid (14:0) pentadecanoic acid (15:0) and heptadecanoic acid (17:0) decreased. Decreased odd chain saturated fatty acids are indicative of decreased food intake at the highest dose, whilst decreased PUFAs reflect differences in lipid oxidation rate where unsaturated lipids are preferentially oxidised with respect to saturated fatty acids (SFA) or MUFAs. Similar results have been reported for PPAR-δ agonists [13].

Overall, PPAR-pan exposure caused a decrease in both essential PUFAs (18:2 *n*−6 and 18:3 *n*−3) indicating an increase in the Δ6 desaturase activity at all doses. Additionally, moderate decreases in *n*−3 and increases in *n*−6 fatty acids were detected, in a dose dependent manner, indicative of liver inflammatory processes. The effects were transitory in nature.

### 3.3 Interconnected signalling networks contribute to liver enlargement and potential DNA damage

The effects of liver enlargement and fat mobilisation as detected by microscopy were further investigated using an LC–MS/MS-based open profiling approach to detect phospholipids (PLs) and triglycerides (TGs) (Supplementary Data 2 [24]). First PLS-DA was used to gain an overview of the intact lipid changes (Fig. 2C). Again, the highest dose group clusters separately, the low to two intermediate dose levels showed a dose dependent effect. In agreement with the microscopic observations the majority of metabolites classified as PLs increased, whereas TGs decreased in concentration (Fig. 2D). Eighty four metabolites were identified as significant, based on their variable impotence in projection (VIP) score which estimates the importance of each variable in the PLS-DA projection. The PLS-DA model variables with VIP≥1 were selected and further characterised based on their fragmentation patterns (Table 3). The report of a general decrease in TG concentrations and an increase in PLs are in agreement with findings describing response to PPAR-δ agonists reported by Roberts and co-workers previously [13] and with findings reported as a response to PPAR-α agonists [19]. In addition, the increases in PL concentrations may potentially provide for the required biomass during membrane development and organelle proliferation of the smooth endoplasmic reticulum (sER) and peroxisomes, effects known to be induced by PPAR-α activation [29]. Since PLs are also natural ligands of LRH2 [30], a nuclear reception known to induce proliferation and hyperplasia [31], it is most likely that LRH2 also contributes to the effects observed to those directly driven by the PPAR-pan agonist GW625019.

**Fig. 3:**
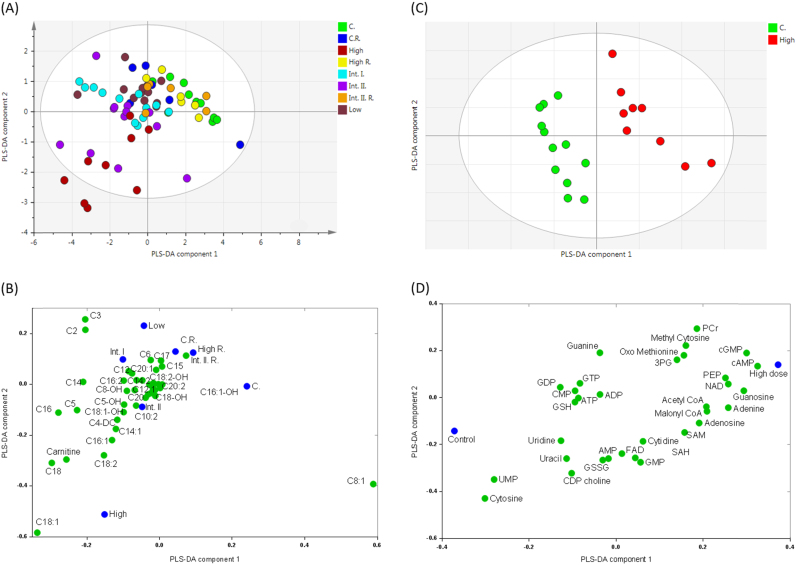
Overview of acyl-carnitine and of aqueous metabolite changes measured in the extracts of liver tissue samples treated with the PPAR-pan agonist. (A) Partial least squares-discriminant analysis (PLS-DA) scores plot and (B) loadings plot of acyl-carnitine changes. (C) PLS-DA scores plot and (D) loadings plot of aqueous metabolite changes. Treatment abbreviations: C., control; Int. I., intermediate 1; Int. II., intermediate 2; R., recovery. Metabolite abbreviations: 3PG, 3-phosphoglycerate; ADP, adenosine diphosphate; AMP, adenosine monophosphate; ATP, adenosine triphosphate; cAMP, cyclic adenosine monophosphate; cGMP, cyclic guanosine monophosphate; CMP, cytidine monophosphate; FAD, flavin adenine dinucleotide; GDP, guanosine diphosphate; GMP, guanosine monophosphate; GSH, glutathione; GSSG, oxidised glutathione; GTP, guanosine triphosphate; NAD, nicotineamide adenine dinucleotide; PCr, phosphocreatine; PEP, phosphoenolpyruvate; SAH, S-adenosyl-homocysteine; SAM, S-adenosyl-methionine; UMP, uridine monophosphate.

Carnitine and acyl-carnitines were measured by LC–MS/MS to investigate whether the decreased TG concentrations are reflected by increased fatty acid β-oxidation (Supplementary Data 3 [24]). Multivariate data analysis was used to generate a PLS-DA model (*R*^2^=87%, *Q*^2^=58%) producing an overview of the changes in carnitine species (Fig. 3A). An increase in the concentrations of all measured carnitines was detected with an exception of octenoyl-carnitine (C8:1) which decreased at all dose levels. Treatment-related increases in the concentrations of free carnitine, acetyl-carnitine (C2), propionyl-carnitine (C3), palmitoyl-carnitine (C4) as well as increased concentrations of stearoyl- (C18:0), oleyl- (C18:1), linoleyl- (C18:2) and palmitoyl- (C16:0) carnitines were observed at all dose levels whilst the recovery groups showed reversibility (Fig. 3B).

Increased concentrations of free- and acyl-carnitine derivatives indicates an increase in β-oxidation, and the profile was the complete opposite to the carnitine profile of hearts from ob/ob mice during high fat substrate excess as reported by Wang et al. [32]. Increased concentration of long-chain acyl-carnitine species could be suggestive of altered long chain 3-hydroxy acyl-CoA dehydrogenase (LCHAD), and very long chain acyl-CoA dehydrogenase (VLCAD) which are likely to be an effect of increased peroxisomal activity reflecting peroxisome proliferation. Another possible source of long-chain acyl-carnitines is TG catabolism, as the fatty acid chains in TG species with decreased concentrations are in agreement with the carnitine increases (both saturated and unsaturated C18 and C16 fatty acids). Accumulation of long-chain carnitines in plasma have also been detected in type 2 diabetes mellitus [33] where incomplete β-oxidation is suggested to be the cause of increased long-chain species, and it has been suggested that overproduction of long chain carnitines can lead to mitochondrial overload and mitochondrial dysfunction [34].

The production of metabolites by oxidation is also exacerbated due to increased mitochondrial and peroxisomal activity. Excessive generation of these metabolites can lead to oxidative stress, DNA damage and eicosanoid production. To study the effects of a discontinued PPAR-pan agonist on oxidative metabolite production the aqueous fractions of liver cell extracts were analysed (Supplementary Data 4 [24]). The highest dose group separated strongly form controls (Fig. 3C), with the discriminatory metabolites related to processes of oxidation and methionine-cycle and 1-carbon metabolism [35]; intermediates such as oxo-methionine, methyl-cytosine and adenosyl-methionine (SAM) concentrations all increased.

In addition nucleic acids cytosine, uridine and uracil were all decreased in concentration following PPAR-pan agonist treatment (Fig. 3D). The sulphur of methionine is particularly prone to oxidation, and forms oxo-methionine, while methionine is also needed to form SAM which serves as a methyl donor in various reactions. Cytosine receives its methyl group from SAM during DNA methylation and in turn SAM forms S-adenosyl-l-homocysteine (SAH). Methyl-cytosine is the main site of methylation for epigenetic modifications, and therefore the increased concentrations of methyl cytosine and SAM induced by the drug treatment suggests epigenetic reprogramming following exposure to the PPAR-pan agonist. Furthermore, increased concentrations of 3-phosphoglycerate (3PG) and phosphoenolpyruvate (PEP), and acetyl- and malonyl-CoA indicate stimulated glycolysis which were shown to be up-regulated in PPAR-δ agonist-treated cells [2]. While malonyl-CoA is known to inhibit CPT-1 in the liver and hence reduce β-oxidation, this does depend on the physiological status of the liver [36], with CPT-1 being partly under the regulation of PPAR-α [37], and thus, malonyl-CoA inhibition of CPT-1 may be overcome by a greater upregulation of the expression of the enzyme.

**Fig. 4:**
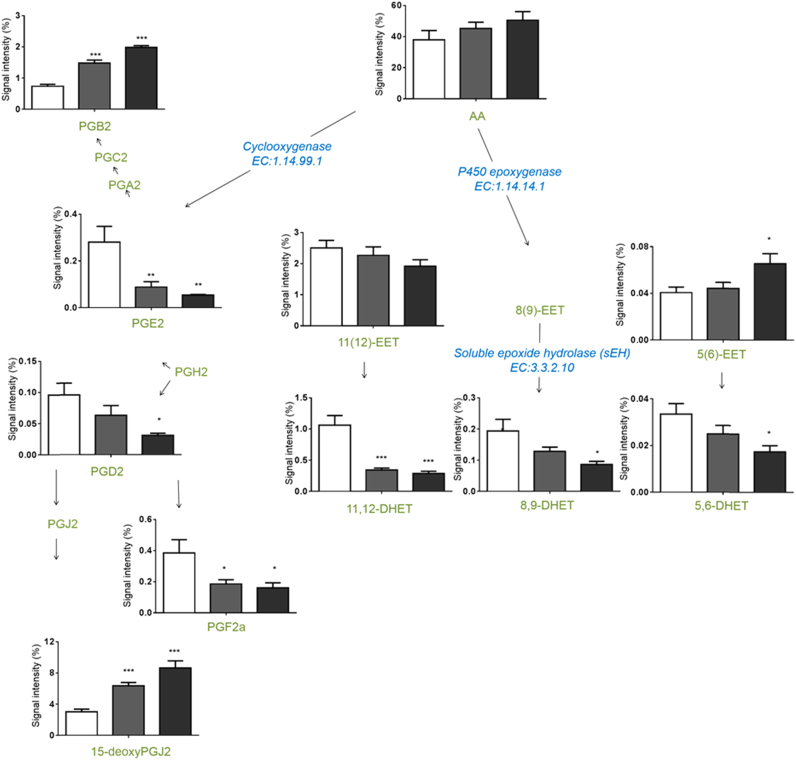
Eicosanoid changes of rat liver tissue samples treated with the PPAR-pan agonist. Bar charts illustrate the changes in relative concentration of those significantly changing species of intermediate dose II (grey) and high dose (black) samples, compared to control (white). Statistical analysis was carried out using one-way analysis of variance (ANOVA) with Bonferoni’s post-test. Values show mean±standard deviation where *n*=12; *p<0.05 **p<0.01***p<0.001. Abbreviations: C., control; AA, arachidonic acid; DHET, dihydroxyeicosatrienoic acid; EET, epoxyeicosatrienoic acid; Int. I., intermediate 1; Int. II., intermediate 2; PG, prostaglandin; R, recovery.

To determine the inflammatory state of the liver caused by this disruption of lipid homeostasis, a range of eicosanoids were measured in the control and the two highest dose level groups (Supplementary Data 5 [24]). A targeted LC–MS/MS approach was used and detected decreases in the concentrations of prostaglandins (PG) PGE2, PGD2, and PGF2 and increases in the concentrations of PGB2 and 15-deoxy PGJ2. Concentrations of dihydroxyeicosatrienoic acids (DHETs) decreased while no significant changes could be measured in the corresponding epoxyeicosatrienoic acid (EET) concentration (Fig. 4). Changes in the concentrations of all measured metabolites are summarised in Table 4.

The PPARs play a critical physiological role as lipid sensors and regulators of lipid metabolism across the whole body. Polyunsaturated fatty acids (PUFAs) such as arachidonic acid and docosahexaenoic acid (DHEA) are endogenous activators of PPARs, linking eicosanoid and related lipid mediator signalling to the PPAR system. High-affinity physiological ligands for the PPARs are currently unknown, but fatty acids and their metabolites can act as activating ligands for PPARs. For example, COX-related metabolites such as prostaglandin D2 (PGD2) and 15-deoxy Δ12,14prostaglandin J2 (15d-PGJ2), activate PPAR-γ [38]. Endogenous activators of PPAR-α include hydroxyeicosatetraenoic acid (8(S)-HETE) and several unsaturated fatty acids, linoleic acid (LA, 18:2 *n*−6), γ-linoleic acid (GLA, 18:3 *n*−6), oleic acid (18:1, *n*−9), as well as arachidonic acid (AA, 20:4 *n*−6). Examples of endogenous PPAR-δ ligands include prostaglandin D2 (PGD2), PGA1 and dihomo-γ-linoleic acid (DGLA, 20:3 *n*−6) [39]. Endogenous PPAR ligands altered by the PPAR-pan agonist include PGE2↓, PGD2↓, PGF2↓, PGB2↑, 15d-PGJ2↑ and DGLA↑ suggesting that these signalling lipids could directly stimulate the PPAR system, despite their low affinity compared to synthetic agonists, because of their high local concentration within the cell. Their low affinity may be compensated by their great variety and their direct synthesis within cells, in favour of achieving intracellular concentrations high enough for receptor mediation.

Other measured changes in eicosanoid levels include decreased concentrations of several dihydroxyeicosatrienoic acids (DHETs) while changes were not detected in the corresponding epoxyeicosatrienoic acids (EETs). EETs are synthesised from AA through epoxygenases, primarily CYP2C and 2J classes in the ER. EETs are also stored in phospholipids, in the sn-2 position of PC,PE and PI and are hydrolysed (much like arachidonic acid) from PLs by phospholipase A2 and released to the extracellular fluid as DHETs [40]. EETs are converted to DHETs in a reaction catalysed by soluble epoxide hydrolase (sEH) which conversion attenuates their anti-inflammatory effects. DHETs are either inactive or minimally active compared with corresponding EETs although DHETs have been found to mediate gene expression levels of both PPAR-α and PPAR-γ [40].

### 3.4 Gene expression changes confirm an increased oxidative environment

To confirm the inflammatory status of the PPAR-pan dosed liver an ELISA assay was carried out profiling 4 cytokines: tumour necrosis factor-alpha (TNF-α), Interleukin 6 (Il-6), interferon production regulator (IFNr) and Interleukin-1 alpha (IL-1α). Four samples were selected from the control, intermediate-2 and the intermediate-2 recovery groups and measured in duplicate using 12 strips each containing 4 different antibodies. The expression of TNFa and Il-1a were further analyse by RT-PCR.

In addition, six substrates were analysed by RT-PCR for representative genes which relate to inflammation (tumour necrosis factor-alpha (TNF-α), Interleukin-1 alpha (Il-1-α)) proliferation (superoxide dismutases-2, SOD2) and oxidative damage (Lon protease-1 (LONp1), Glutathione-S-transferase-kappa-1 (GST-κ-1), glutathione synthase (GSH synthase)).

**Fig. 5:**
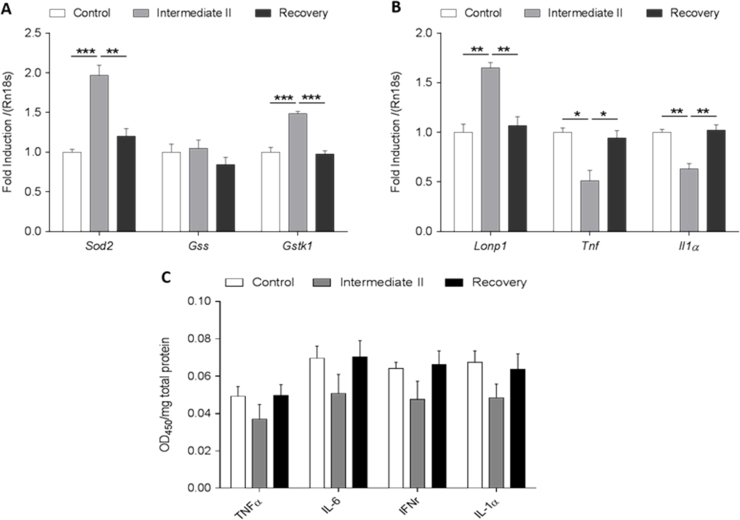
The effects of PPAR-pan induction in the expression of proliferative (superoxide dismutases-2, SOD2), oxidative damage ((Glutathione-S-transferase-kappa-1 (GST-κ-1), glutathione synthase (GSH synthase), Lon protease-1 (LONp1)), and inflammatory (tumour necrosis factor-alpha (TNF-α), Interleukin-6 (Il-6), interferon production regulator (IFNr) and Il-1-α) target genes and/or proteins in rat liver tissues. (A) Increased expression of SOD2, GST-κ-1 and (B) increased expression of Lonp-1 was detected, whereas levels of TNF-α and Il1-α mRNA expression decreased as measured by real time quantitative PCR assay. We used 18 s as a control for RNA loading. (C) PPAR-pan dependent inhibition of TNF-α, Il-6, IFNr and Il-1-α protein expression in determined by ELISA assay.

Our results show an increase in the markers of oxidative damage markers Lon protease (LONp), Glutathione-S-transferase-kappa-1 (GST-κ-1), whilst no change was detected in GSH synthase expression (Fig. 5A), and cytokines showed a reduction in the dosed group samples at both the RNA and protein levels (Fig. 5B and C).

Our results indicate an environment, where oxidative damage is increased which is in agreement with metabolomics and lipidomics findings, whereas levels of cytokines were found to decrease. It has been reported, that certain PPAR-γ ligands, especially 15-deoxyPGJ2, antagonise the expression of TNF-α and IL-6 in response to macrophage activation [41], [42]. Furthermore, the administration of PPAR-α agonists have been shown to reduce the activity of nuclear factor-κB (NF-κB) leading to a loss in cytokine production [43]. It is also widely accepted, that oxidative stress induced activity promotes the production of a number of pro-inflammatory cytokines, which can contribute to the pathology of many disease states [44]. The dichotomy between the increase in several oxidative stress markers (LONp, GST-κ-1 oxo-methionine, methyl-cytosine, SAM, acyl-carnitines, and eicosanoids) and the decrease of inflammatory cytokine markers points to additional signalling pathways which contribute to the regulation of inflammatory metabolite production. Our results indicate, that an important consequence of PPAR-pan activation may be functioning to limit the production of inflammatory mediators in the presence of increased oxidation. Reducing inflammation is likely to limit the production of inflammatory molecules (including several oxidised lipids and pro-inflammatory eicosanoids) which would further exacerbate hypertrophy and increase hepatic leakage enzyme levels in the plasma such as ALT and AST.

## 4 Conclusions

In this study we have applied a combined metabolomic and lipidomic screen to investigate changes induced by a discontinued PPAR-pan agonist in male SD rat livers. The livers of treated animals exhibited a classic dose-dependent phenotype indicative of sub-cellular organelle proliferation, including increased weight, hepatocyte eosinophilia and hypertrophy. Mechanistic studies have previously described the observed hypertrophy as a consequence of proliferation of peroxisomes and smooth ER, due to the combined activation of PPAR-α and PPAR-δ.

From the work described here, the proliferation is in part associated with alterations in the eicosanoid profile. While stimulation of PPAR-α and PPAR-δ both increase fatty acid oxidation, reducing triglycerides which might be seen as a favourable response, the agonist also increases the production of metabolites related to processes of oxidation which are associated with inflammation and DNA damage. In particular several inflammatory eicosanoids (prostaglandins) are altered in concentration.

## Figures and Tables

**Table 1:** Study design.

Group description	Dose (mg/kg/day)	Animal number	Recovery animals

Control	0	1–12	13–18
Low	30	19–30	–
Intermediate 1	100	31–42	–
Intermediate 2	300	43–54	55–60
High	1000	61–72	73–78

**Table 2:** Clinical chemistry parameters. Abbreviations: ALP, alkaline phosphatise; ALT, alanine aminotransferase; AST, aspartate aminotransferase; Bil, total bilirubin; CK, creatine kinase.

Parameters	Dose groups (mg/kg/day)				
	0	30	100	300	1000

ALT (IU/L)	74±14	71±15.6	77±23.1	117±10.38	116±58.7
AST (IU/L)	115±14.2	112±15.1	105±15.2	295**±46.28	257***±87.6
ALP (IU/L)	303±66.4	314±72.8	380*±79.9	429***±105.4	815***±154
Bil (umol/L)	2.6±0.55	1.0±0.33	1.0***±0	1.0***±0.25	5.7*±4.66
Albumin (g/L)	46±2.7	50*±4.7	53***±3.6	53***±5.1	50±2.9
Glucose (mmol/L)	4.4±0.8	4.3±0.49	4.9±0.79	4.8±0.68	5.7±0.68*
CK (g/L)	64±3.5	62±3.3	64±3.5	62±4.9	58***±4.2

**Table 3:** Changes in phospholipids and triacylglycerols with variable importance in projection (VIP) score equal or greater than one comparing the control to the high dose group samples. The arrows indicate the direction of the chance. Abbreviations: LPC, lyso-phosphocholine; PC, phosphocholine; PE, phosphoethanolamine; RT, retention time; TG, triacylglycerol; VIP, variable importance in projection.

RT	*m/z*		VIP score	ID	
1.04	544.332	↓	1.1	LPC(20:4)	LPC(20:4)
1.31	426.353	↓	1.1	LPE(14:0)	LPE(14:0)
6.76	808.584	↓	1.2	PC(38:5)	
6.92	808.584	↓	1.7	PC(38:5)	
7.24	784.584	↓	1.7	PC(36:3)	PC(18:1_18:2)
7.41	766.538	↓	2.0	PE(38:5)	
8.85	832.581	↓	1.1	PC(40:7)	PC(20:4_20:3)
8.86	810.600	↓	3.5	PC(38:4)	
9.23	808.580	↓	1.1	PC(38:5)	PC(18:0_20:5)
9.24	786.600	↓	3.7	PC(36:2)	PC(18:1/18:1)
9.26	718.535	↓	1.4	PE(34:1)	
13.08	813.683	↓	1.5	PC(37:4)	
13.18	638.569	↓	1.2	DG(36:2)	
13.19	643.525	↓	1.8	DG(38:5)	
13.42	801.682	↓	1.4	PC(36:3)	PC(18:1_18:2)
13.74	749.545	↓	1.2	TG(37:3)	
14.49	818.613	↓	1.5	TG(48:3)	
14.49	795.627	↓	1.8	TG(48:6)	
14.50	812.653	↓	3.3	TG(48:6)	
15.28	875.706	↓	1.1	TG(54:8)	TG(18:2_16:0_20:6)
15.34	870.750	↓	1.5	TG(52:5)	TG(18:3_18:2_16:0)
15.35	920.766	↓	1.2	TG(56:8)	TG(18:2_16:0_22:6)
15.47	896.767	↓	2.5	TG(54:6)	TG(18:1_18:2_18:3)
15.48	902.726	↓	1.4	TG(54:3)	TG(20:1_20:1_14:1)
15.49	922.782	↓	1.9	TG(56:7)	TG(16:1_20:4_20:2)
15.49	928.742	↓	1.1	TG(57:11)	TG(20:0_18:0_18:4)
15.57	898.782	↓	2.2	TG(54:5)	TG(20:3_18:2_16:0)
15.59	878.727	↓	2.1	TG(53:8)	TG(18:0_20:1_14:0)
15.59	872.768	↓	4.1	TG(52:4)	TG(18:2_18:2_16:0)
15.75	898.783	↓	1.5	TG(54:5)	TG(20:3_18:2_16:0)
15.76	695.571	↓	1.0	TG(40:0)	TG(24:0_6:0_10:0)
15.82	848.768	↓	2.5	TG(50:2)	TG(16:0_16:0_18:2)
15.85	874.785	↓	2.0	TG(52:3)	TG(16:0_18:2_18:1)
15.86	900.799	↓	1.4	TG(54:4)	TG(18:1_18:1_18:3)
15.96	666.616	↓	1.1	DG(38:2)	DG(20:0_18:2)
15.96	671.572	↓	1.1	DG(40:5)	
16.12	902.815	↓	1.1	TG(54:3)	TG(18:1_18:1_18:1)
16.39	904.830	↓	1.6	TG(54:2)	TG(18:1:18:1_18:0)
16.39	884.774	↓	1.4	TG(53:5)	
16.39	878.816	↓	2.9	TG(52:1)	TG(16:0_18:0_18:1)
16.39	884.774	↓	1.4	TG(53:5)	
1.79	622.401	↑	1.0	PC(24:0)	
3.41	790.550	↑	1.5	PC(37:7)	
5.24	830.567	↑	1.8	PC(40:8)	
6.68	804.550	↑	2.2	PC(38:7)	
6.98	780.550	↑	2.2	PC(36:5)	
7.21	740.522	↑	4.0	PE(36:4)	PE(22:4_14:0)
8.35	784.584	↑	1.9	PC(36:3)	
8.66	760.585	↑	10.5	PC(34:1)	
8.66	782.566	↑	3.4	PC(36:4)	PC(16:0_20:4)
8.82	782.566	↑	1.2	PC(36:4)	PC(16:0_20:4)
8.84	760.585	↑	3.0	PC(36:4)	PC(18:1_18:3)
9.54	768.552	↑	3.7	PE(38:4)	
9.77	768.553	↑	1.4	PE(38:4)	
11.45	788.615	↑	2.9	PC(36:1)	PC(18:1_18:0)
11.74	788.616	↑	1.4	PC(36:1)	PC(18:1_18:0)
13.13	577.518	↑	1.1	DG(33:3)	
13.14	617.510	↑	1.7	DG(36:4)	
13.61	647.457	↑	1.8	TG(37:3)	
13.81	670.608	↑	1.3	TG(37:0)	
14.35	672.625	↑	1.2	DG(39:6)	
14.50	833.582	↑	1.2	TG(51:8)	
14.50	817.609	↑	1.7	TG(50:9)	
15.81	854.726	↑	1.3	TG(51:6)	
16.11	850.784	↑	2.1	TG(50:1)	TG(16:0_16:0_18:1)
16.11	876.800	↑	9.5	TG(52:2)	TG(16:0_18:1_18:1)
16.11	882.759	↑	2.9	TG(53:6)	

**Table 4:** Relative concentration changes in the measured eicosanoids. Significant changes as determined by ANOVA with Bonferoni’s post test for multiple comparisons labelled ↑*p*<0.05, ↑↑ *p*<0.01, ↑↑↑ *p*<0.001 for increased and ↓*p*<0.05,↓↓ *p*<0.01, ↓↓↓ *p*<0.001 (*n*=12). Abbreviations: AA, arachidonic acid; DGLA, dihomo-γ-linolenic acid; DHEA, docosahexaenoic acid; DHET, dihydroxyeicosatrienoic acid; DHOME, dihydroxyoctadecenoic acid; EET, epoxyeicosatrienoic acid; HDoHE, hydroxydocosahexaenoic acid; HEPE, hydroxyeicosapentaenoic acid; HETE, hydroxyeicosatetraenoic acid; HODE, hydroxyoctadienoic acid; LT, leukotriene; ODE, octadienoic acid, PG, prostaglandin; THET, trihydroxyeicosatetraenoic acid; THF, tetrahydrofuran; TX, thromboxane.

		Inter-mediate 2	High dose			Inter-mediate 2	High dose
1	11(12)-EET	―	―	24	8,9-DHET	―	↓
2	11,12,15-THET	―	↑↑↑	25	8-HETE	―	―
3	11,12-DHET	↓↓↓	↓↓↓	26	8-iso-PGE2	↓↓	↓↓
4	11-HEPE	↓↓↓	↓↓	27	8-isoPGF2a	↓	↓↓
5	11-HETE	―	―	28	9(10)-EpOME	↓	↓↓
6	12(13)-EpOME	↓↓	↓↓	29	9,10,13-TriHOME	―	―
7	12,13-DHOME	↓↓↓	↓↓	30	9,10-DHOME	↓↓↓	↓↓↓
8	12-HEPE	↓↓↓	↓↓↓	31	9,12,13-TriHOME	―	↓
9	12-HETE	―	↓↓	32	9-HODE	↓	↓↓
10	13-HDoHE	↓	↓↓	33	9-oxo-ODE	↓	↓↓
11	13-HODE	↓	↓↓	34	AA	―	―
12	13-oxo-ODE	―	↓	35	DHEA	―	―
13	14(15)-EET	―	―	36	DGLA	↑	↑↑↑
14	14,15-DHET	―	―	37	Lipoxin A4	―	↓
15	15-deoxyPGJ2	↑↑↑	↑↑↑	38	LTB4	―	―
16	15-HETE	―	―	39	PGB2	↑↑↑	↑↑↑
17	15-oxo-EET	―	―	40	PGD2	―	↓
18	19-HETE	―	―	41	PGE2	↓↓	↓↓
19	20-HETE	―	―	42	PGF2a	↓	↓
20	5(6)-EET	―	↑	43	THF diols	―	―
21	5,6-DHET	―	↓	44	TXB2	―	―
22	5-oxo-EET	―	―				

## Footnotes

Appendix A Supplementary data associated with this article can be found in the online version at 10.1016/j.freeradbiomed.2015.11.033.
