# Supplementary material for: PPAR-pan activation induces hepatic oxidative stress and lipidomic remodelling
Source: Free Radic Biol Med. 2016 Jun;95:357–68. doi: 10.1016/j.freeradbiomed.2015.11.033 (PMC4891066; doi:10.1016/j.freeradbiomed.2015.11.033)
Supplement: Supplementary file 1 [file mmc1.pdf]

Supplementary table 1: Summary of microscopy findings according to treatment groups.
Abbreviations: TK, terminal kill; R, recovery.

			Control	Low dose	Inter-mediate 1	Inter-mediate 2	High
Muscle	myopathy/ inflammation	TK	7/12	5/12	7/12	9/12	6/9
		R	6/6			6/6	3/6
Liver	hepatocyte vacuolation	TK			1/12		
		R					
	Hypertrophy	TK	0/12	5/12	12/12	12/12	9/9
		R				2/6	2/6
Heart	myopathy/ inflammation	TK	4/12	4/12	5/12	8/12	9/9
		R	5/6			2/6	4/6
Mesenteric fat	reduced limphocyte size	TK					3/9
		R					
Adrenal gland	increased microvacuolation	TK					5/9
		R					2/6
Thyroid gland	Hypertrophy	TK	1/12	2/12	1/12	1/12	2/9
		R					
Diaphragm	Myopathy	TK				2/12	8/9
		R					2/6
Gastrocnemius muscle	Myopathy	TK	1/12	2/12	1/12	3/12	8/9
		R					
Soleus muscle	Myopathy	TK	4/12	7/12	11/12	10/12	8/9
		R	1/6				
Intercostal muscle	Myopathy	TK	0/12	1/12	0/12	5/12	9/9
		R					
Brown subcut fat	macrovacuolation	TK	2/12	1/12	2/12	4/12	5/9
		R				1/6	1/6
Aortic brown fat	macrovacuolation	TK	0/12	3/12	6/12	4/12	4/9
		R	1/6				1/6
Epididymal fat	Steatitis	TK			1/12		
		R					

Supplementary table 2: A list of mass to charge (*m/z*) ratios showing $[M\text{-}H]^-$ values for each measured aqueous metabolite and the corresponding fragment ion after collision-induced dissociation (CID). Q1 denotes the parent ions, whereas Q3 denotes the fragment ions. **Abbreviations:** 3PG, 3-phosphoglycerate; ADP, adenosine diphosphate; AMP, adenosine monophosphate; ATP, adenosine triphosphate; cAMP, cyclic adenosine monophosphate; cGMP, cyclic guanosine monophosphate; CMP, cytidine monophosphate; FAD, flavin adenine dinucleotide; GDP, guanosine diphosphate; GMP, guanosine monophosphate; GSH, glutathione; GSSG, oxidised glutathione; GTP, guanosine triphosphate; NAD, nicotineamide adenine dinucleotide; PCr, phosphocreatine; PEP, phosphoenolpyruvate; SAH, S-adenosyl-homocysteine; SAM, S-adenosyl-methionine; UMP, uridine monophosphate.

Analyte	Q1	Q3	Analyte	Q1	Q3
3-PG	187.0	105.0	GSH	308.1	179.0
Acetyl-CoA	810.0	303.2	GSSG	613.1	355.0
Adenine	136.0	119.0	GTP	523.9	152.0
Adenosine	268.1	136.1	Guanine	152.0	134.9
ADP	428.0	136.0	Guanosine	284.1	152.1
AMP	348.0	136.0	Malonyl-CoA	854.0	347.1
ATP	508.0	136.0	Methyl-cytosine	126.0	109.1
cAMP	330.1	136.1	NAD	664.0	427.9
CDP-choline	489.1	184.1	Oxo-methionine	165.0	105.0
cGMP	346.1	152.1	PCr	212.1	177.1
CMP	324.1	112.0	PEP	169.1	150.9
Cytidine	244.1	112.0	SAH	385.1	136.1
Cytosine	112.0	95.0	SAM	399.0	250.1
FAD	786.1	348.0	UMP	325.1	96.9
GDP	444.0	152.0	Uracil	112.9	70.1
GMP	364.2	152.1	Uridine	245.1	112.9

Supplementary table 3: Eicosanoid method gradient

Analysis		
column	C_{18} Luna column (Phenomenex)	
column dimensions	150 mm x 2 mm, 3 µm	
Liquid chromatograph	Waters Acquity	
flow rate	0.4 mL/min	
mobile phase A	0.1 % acetic acid	
mobile phase B	0.1 % acetic acid	
	in acetonitrile: methanol (80 :20)	
gradient	0 min	15% B
	1.5 min	30% B
	10.5 min	60% B
	16 min	80% B
	19 min	100% B
	19.1 min	15% B
	21 min	15% B

Supplementary table 4: A list of mass to charge (*m/z*) ratios showing $[M-H]^-$ values for each measured eicosanoid and the corresponding fragment ion after collision-induced dissociation (CID). Q1 denotes the parent ions, whereas Q3 denotes the fragment ions **Abbreviations**: AA, arachidonic acid; DGLA, dihomo-γ-linolenic acid; DHEA, docosahexaenoic acid; DHET, dihydroxyeicosatrienoic acid; DHOME, dihydroxyoctadecenoic acid; EET, epoxyeicosatrienoic acid; HDoHE, hydroxydocosahexaenoic acid; HEPE, hydroxyeicosapentaenoic acid;
HETE, hydroxyeicosatetraenoic acid; HODE, hydroxyoctadienoic acid; LT, leukotriene; ODE, octadienoic acid, PG, prostaglandin; THET, trihydroxyeicosatetraenoic acid; THF, tetrahydrofuran; TX, thromboxane.

Analyte	Q1	Q3	Analyte	Q1	Q3
11(12)-EET	319.2	167	8,9-DHET	337.2	127.1
11,12,15-THET	353.2	167.1	8-HETE	319.2	301.2
11,12-DHET	337.2	167.1	8-iso-PGE_2	351	271
11-HEPE	317	169	8-isoPGF_2α	353.1	193.2
11-HETE	319.18	166.9	9(10)-EpOME	295.2	171.1
12(13)-EpOME	295.2	195.2	9,10,13-TriHOME	329.2	171.1
12,13-DHOME	313.2	183.2	9,10-DHOME	313.2	201.2
12-HEPE	317.17	179	9,12,13-TriHOME	329.2	211.1
12-HETE	319.2	179.2	9-HODE	295.2	171
13-HDoHE	343.13	193	9-oxo-ODE	292.2	185.1
13-HODE	295.2	195	AA	303.3	259.1
13-oxo-ODE	239.2	113	DHEA	327.2	283.1
14(15)-EET	319.2	219.3	DGLA	305.19	58.8
14,15-DHET	337.19	206.9	Lipoxin A4	351.2	115.2
15-deoxyPGJ2	315.2	271.3	LTB_4	335.2	195.1
15-HETE	319.2	301.4	PGB_2	333.069	59
15-oxo-EET	317.2	113.1	PGD_2	351.2	271.3
19-HETE	319.2	275.1	PGE_2	351.2	271.3
20-HETE	319.2	275.2	PGE_2-d_4	355.3	275.3
5(6)-EET	319.2	191	PGF_2α	353.2	309.3
5,6-DHET	337.2	145.1	THF diols	353.2	167.1
5-oxo-EET	317.2	273.2	TXB_2	369.2	169.1
